# Intravascular ultrasound assisted sizing in thoracic endovascular aortic repair improves aortic remodeling in Type B aortic dissection

**DOI:** 10.1371/journal.pone.0196180

**Published:** 2018-04-19

**Authors:** Julia Lortz, Konstantinos Tsagakis, Christos Rammos, Michael Horacek, Thomas Schlosser, Heinz Jakob, Tienush Rassaf, Rolf Alexander Jánosi

**Affiliations:** 1 Department of Cardiology and Vascular Medicine, West-German Heart and Vascular Center Essen, University of Duisburg-Essen, Essen, Germany; 2 Department of Thoracic and Cardiovascular Surgery, West-German Heart and Vascular Center Essen, University of Duisburg-Essen, Essen, Germany; 3 Department of Diagnostic and Interventional Radiology and Neuroradiology, University Hospital Essen, University of Duisburg-Essen, Essen, Germany; Temple University School of Medicine, UNITED STATES

## Abstract

The precise sizing of the stent graft in thoracic endovascular aortic repair (TEVAR) affects aortic remodeling and hence, further outcome. Covering the proximal entry tear is essential for successful treatment of Type B aortic dissection. Intravascular ultrasound (IVUS) enables real-time aortic diameter assessment, and is especially useful when computed tomography (CT) image quality is poor. IVUS, however, is not routinely utilized due to cost inefficiency. We investigated the impact of IVUS-assisted stent graft sizing on aortic remodeling in TEVAR. In this single-center retrospective study we evaluated patients with Type B aortic dissection undergoing both CT and IVUS before TEVAR. We assessed the aortic diameter at the level of the left subclavian artery via both methods before stent implantation and analyzed due to which method the implanted stent graft was chosen, retrospectively. To determine the degrees of aortic remodeling involved, we evaluated true lumen and false lumen diameters, and total aortic remodeling in CT. We analyzed 45 patients with Type B aortic dissection undergoing TEVAR. The mean ages were 66.9±10.0 years fo0072 IVUS (n = 20) and 62.3±14.2 years for CT-assisted TEVAR (n = 25; p = 0.226). The follow-up time for both groups did not differ between the two groups (IVUS: 22.9±23.1 months, CT: 25.6±23.0 months; p = 0.700). While both methods were associated with advantages regarding aortic remodeling, IVUS-assisted sizing yielded a greater increase in true lumen (30.4±6.2 vs. 25.6±5.3; p = 0.008) and reductions in false lumen (14.4±8.5 vs. 23.9±9.3; p = 0.001) and total aortic diameter (35.5±6.0 vs. 39.9±8.1; p = 0.045). IVUS-guided stent graft sizing in Type B aortic dissection shows beneficial effects on aortic remodeling and might be of additional advantage in aortic diseases, especially when CT image quality is poor.

## Introduction

The development of thoracic endovascular aortic repair (TEVAR) represents a cornerstone in the current treatment of the acute aortic syndrome. In the treatment of uncomplicated Type B aortic dissection, medical therapy remains the state of the art therapy [1,2]. But recent studies have yielded promising results with regard to disease progression and aortic remodeling and suggest a change in paradigm in favor of TEVAR [3,4]. As TEVAR gains increasing significance, its optimal application is the key to the successful treatment of Type B aortic dissection by covering the proximal entry tear. The correct size and placement of the aortic stent graft has strong effects on remodeling, re-intervention rate, and subsequent outcome of patients [5–7]. Computed tomography (CT) is still the state-of-the-art technique for diagnosis and the planning of further treatment in most aortic diseases, which is at least partly due to its widespread availability. As CT is generally performed at the time of diagnosis, there is often a timely delay between CT and TEVAR, leading to differences in the measured diameters due to changes in hemodynamic and volume filling, *e*.*g*., in patients with aortic rupture the associated hemodynamic compromise can affect intravascular volume and the real diameters may be underestimated. Furthermore, in cases where CT images lack reliable imaging quality, choosing the correct stent graft can be challenging. Intravascular ultrasound (IVUS) closes this time-gap using real-time characterization of the lesion and relevant diameters including intraluminal assessment [8], but it is not routinely utilized due to cost inefficiency and a relative lack of availability. Moreover, the benefits of IVUS-guided graft sizing of landing zones in emergency TEVAR in Type B aortic dissection have not yet been conclusively evaluated. The current study investigated whether IVUS-guided stent sizing influences aortic remodeling over time.

## Materials and methods

### Study design and patient allocation to sizing strategy

In a single-center, prospective study we evaluated the impact of IVUS-assisted stent sizing in patients undergoing TEVAR using aortic stent prostheses. All patients who received TEVAR from October 2006 to November 2016 at our institution were examined. The indications for treatment included aortic rupture with hemothorax and/or hemomediastinum, persistent pain, refractory hypertension, acute limb, intestinal, or renal ischemia, a maximum descending or thoracic aortic diameter of 40 mm (acute aortic dissection) or 50 mm (chronic aortic dissection). Those with Type B aortic dissection and both IVUS and CT before TEVAR were identified and clinical data including IVUS and CT measurements obtained. Aortic dissection was considered to be acute within the first 14 days from symptom onset, whereas after 14 days it was considered to be chronic [1]. All patients were divided into two groups depending of the used sizing strategy used within the TEVAR procedure by independent cardiologists (IVUS-preferred vs. CT-preferred). The assessed IVUS and CT measurements pre-treatment were compared to the used stent graft size used and manually analyzed on the basis of which measurement was chosen.

**Fig 1:**
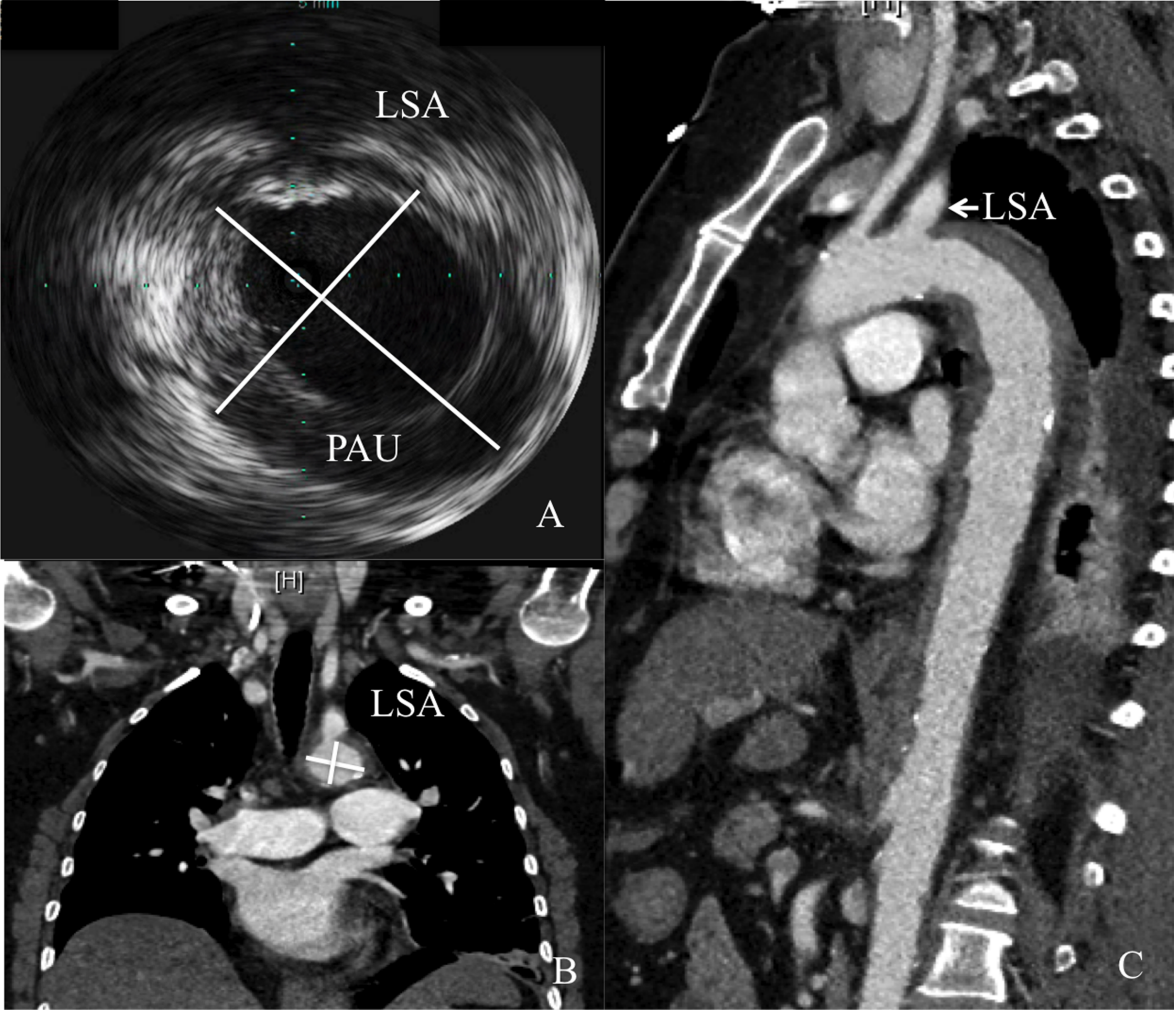
Example for measurement of the aortic diameter at level of the left subclavian artery (LSA) with Type B aortic dissention (+). CT (A) and IVUS (B) measurements at the level of the LSA (*). Penetraiting aortic ulcer (PAU) is shown in IVUS.

We measured aortic diameter (AD) at the level of the left subclavian artery (LSA) in IVUS and CT as a benchmark for measurements and further proximal landing zone for the definite stent alignment before stent implantation (Fig 1). Minimum and maximum diameter were measured and electrography-traced in both IVUS and CT to obtain mean diameter. The definite stent graft choice was made in consultation with another experienced cardiologist who had no prior knowledge of the cases. All patients were treated at a major tertiary referral center by a team of interventional cardiologists, cardiothoracic surgeons, and anesthesiologists with significant expertise in acute aortic syndromes. Due to the retrospective design of the current study the assessment of aortic diameters and further stent choice were done independently. Only patients with a defined proximal landing zone at the level of the LSA were included. The study was approved by the local ethics committee of the University of Duisburg-Essen and was conducted in accordance with the Declaration of Helsinki. Patient records were de-identified and analyzed anonymously. Therefore, the local ethics committee approved the retrospective analysis of the patient data without the need to obtain patient consent.

### IVUS procedure and following TEVAR

IVUS studies were performed at the time of TEVAR using the Visions PV 0.035 catheter system (Volcano, San Diego, CA, USA). The IVUS catheter uses 10-MHz frequency ultrasound and has a maximum imaging diameter of 60 mm. A pigtail catheter was inserted into the ascending aorta. Over a long guidewire that was introduced over the catheter, the IVUS catheter was positioned in the ascending aorta close to the aortic valve. The IVUS probe was manually retracted to a point corresponding to the distal part of the sheath. Grayscale images were simultaneously acquired digitally. Gyrating movements were used in an attempt to gain an optimal cross-sectional aortic image. The cardiologist performing these IVUS scans had previous experience acquiring IVUS measurements from more than 50 aortas. The time of definite TEVAR was dependent on underlying aortic disease, and was divided into elective and emergency TEVAR procedures. The following stent grafts were used: Valiant and Valiant-Captivia (Medtronic, Minneapolis, MN, USA), Relay (Bolton Medical, Barcelona, Spain), and GORE TAG (W.L. Gore & Ass., Flagstaff). The chosen stent graft size included 10% of oversizing (Medtronic, Bolton Medical) or was obtained from the range of the manufacturer’s guidelines (GORE). The TEVAR procedure was performed in the cardiac catheterization laboratory by a team of interventional cardiologists, cardiothoracic surgeons, and anesthesiologists under general anesthesia receiving mechanical ventilation. The procedure was performed under sterile conditions and intravenous antibiotics were administered prior (ceftriaxone). After surgical exposure of the femoral artery, a standard 6-French arterial sheath was administered and 5000|IU of heparin were injected. An angiogram was obtained using a graduated 6-French pigtail catheter with metallic markers to determine the anatomical conditions, including location of side branches (i.e. LSA) and develop the strategy for the optimal stent-graft landing zones. Additionally, a 6-French pigtail catheter was advanced from the left radial artery to enable intra-procedural angiography. The stent-graft delivery system was advanced over an ultrastiff 0.035|in guidewire (Meier Back-Up, Boston Scientific, Oakland, CA, USA). The stent-grafts were positioned in the thoracic aorta under fluoroscopic guidance. Before the stent-graft was deployed, systolic blood pressure was lowered to ∼50|mmHg using intravenous sodium nitroprusside to prevent inadvertent downstream displacement of the stent-graft during delivery. Immediate procedural success was evaluated via angiography. No additional heparin or antiplatelet medications were administered following completion of the procedure.

### CT study

Contrast-enhanced, electrocardiogram-triggered, high-resolution (<_1.5mm slice thickness) CT angiography (CTA) was performed either on a 64-row multidetector scanner at our institution (Somatom Definition; Siemens Healthcare, Forchheim, Germany) or were done already at the referring outside facility. The following examination protocol is referred to our institution. Continuous scans covering the entire aorta, including the proximal supraaortic vessels down to the groin. Iodinated contrast (120 to 140 mL) was continuously injected into the right antecubital vein via an 18-G catheter at an infusion rate of 3.5 mL/s. To ensure maximum contrast concentration in the aorta, a circular region of interest (ROI) was placed in the ascending aorta. As soon as the signal intensity in the ROI reached a threshold of 120 Hounsfield units, the patient was instructed to maintain an inspiration breath hold, at which point data acquisition commenced. A second late arterial phase scan was performed after a delay of 15 s, covering the same area. Imaging was performed prior to TEVAR, the day after TEVAR, and at a follow-up time after an unspecified time (non-standardized) interval.

### Aortic remodeling

**Fig 2:**
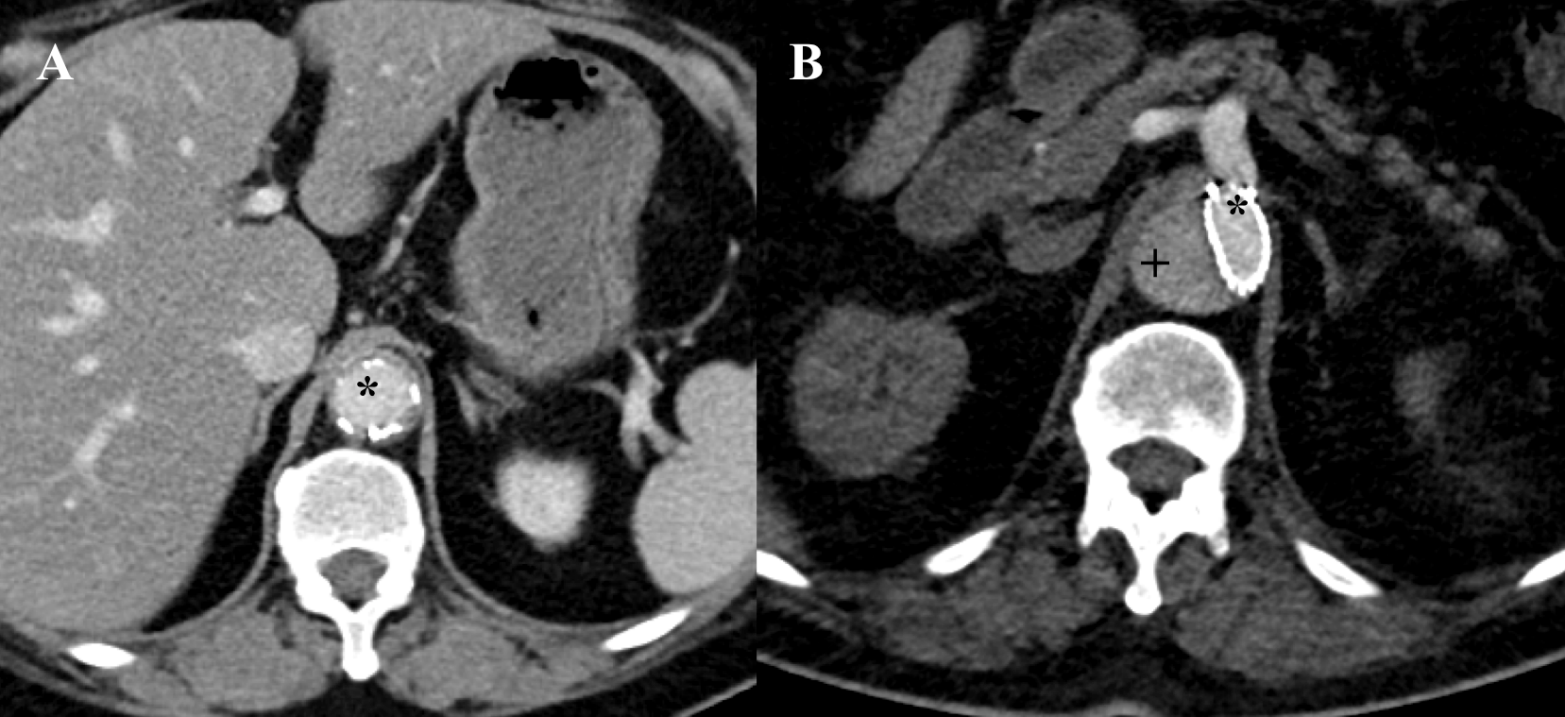
Example for assessment of aortic remodeling at the inferior edge of the stentgraft with sufficient remodeling (A) and impaired remodeling (B). A: True lumen (*) is fully expanded, no false lumen is definable. B: False lumen (+) represents almost two-thirds of total aortic area at this intersection.

For the assessment of the aortic remodeling, we analyzed CT examinations from the Department of Radiology at our institution and performed the assessments as previously described. The CT procedure that was performed the day after TEVAR (post-treatment) served as benchmark for locating the level of the inferior edge of the stent graft. The minimum and maximum diameters of the true lumen (TL), false lumen (FL), and total AD were assessed in the pre-treatment, post-treatment and follow-up CT scans, and mean diameters were calculated (Fig 2). An additional cross-sectional analysis was done at the level of the pulmonary artery and the diaphragm in the follow-up CT scans. For follow-up values, we evaluated the most recently acquired CT scans. All measurements were made by two independent observers in a blinded manner.

### Statistical analysis

Continuous variables are presented as means ± standard deviation, while categorical variables are presented as frequencies and percentages. The Mann-Whitney U test and chi-square test were used for the comparison of categorical variables. Student’s *t*-test was used for the analysis of continuous variables. Values of p < 0.05 were considered statistically significant. All data and statistical analyses were performed using SPSS 24 (Chicago, IL, USA) for Mac and Microsoft Excel 2011 for Mac.

## Results

### Study population

A total of 45 patients with Type B aortic dissection received TEVAR between October 2006 and November 2016 and underwent previous IVUS and CT studies. Twenty patients received a stent graft based on IVUS measurements, and 25 received a stent graft based on CT. One case was added to the CT group because the sized stent graft for both methods would have been the same independent of sizing strategy, thus IVUS was not superior. The relevant patient demographics are shown in Table 1. Although no relevant differences in comorbidities were found between the groups, IVUS-preferred sizing was more often used in acute aortic dissections compared to CT.

**Table 1:** Patient demographics.

	IVUS *n* = 20	CT *n* = 25	*p* value
Age, years, *mean±SD*	66.9±10.0	62.3±14.2	0.226
Men, *n* (%)	10 (50)	10 (40)	0.507
Acute aortic dissection, *n* (%)	19 (95)	13 (52)	0.002*
IMH, *n* (%)	3 (15)	6 (24)	0.453
Emergency procedure	15 (60)	4 (16)	0.006*
Coronary artery disease, *n* (%)	9 (45)	8 (32)	0.337
Hypertension, *n* (%)	19 (95)	21 (84)	0.249
Renal impairment, *n* (%)	6 (30)	7 (28)	0.884
Diabetes, *n* (%)	5 (25)	2 (8)	0.122
Smoking, *n* (%)	12 (60)	10 (40)	0.187
Previous aortic surgery, *n* (%)	4 (20)	4 (16)	0.730

IMH, intramural hematoma; IVUS, intravascular ultrasound; CT, computed tomography.

* means significant

### Comparison of sizing methods and used stent grafts

**Fig 3:**
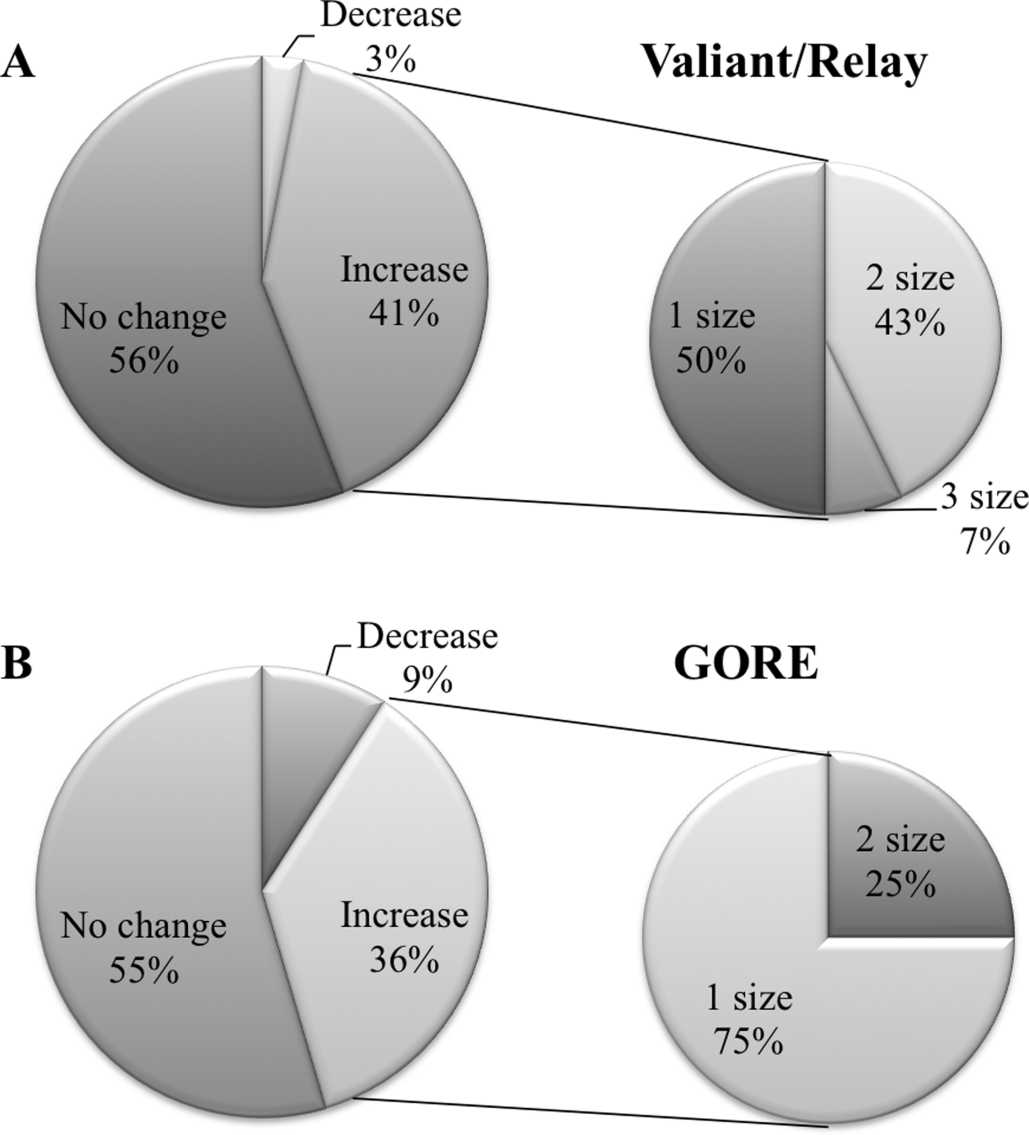
In more than 40%, intravascular ultrasound (IVUS) leads to a different sizing strategy in aortic stent grafts. Shown are the distributions for changes in the sizing strategy made by IVUS in Valiant/Relay stent grafts (A) and GORE according to the recommended sizing chart (B). Further subdivision of the increases in stent graft sizes is presented in the smaller, right circles.

In comparisons of IVUS and CT measurements, there was a significant difference between both methods in the collective group (IVUS: 33.8±3.7 mm, CT: 31.4±5.8; *p* = 0.030). This difference was still significant after allocation: The IVUS-preferred group showed larger diameters in IVUS measurements compared to CT (IVUS: 34.4±3.1 mm, CT: 31.6±6.4; *p* = 0.045); the same observation was made in the CT-preferred group (IVUS: 33.3±4.1 mm, CT: 31.2±5.4; *p* = 0.037). IVUS measured diameters between both groups did not differ (p = 0.347), likewise for CT measured diameters (p = 0.801). The distribution of the stent grafts used is shown in Table 2. The implanted stent grafts chosen based on CT measurements were smaller than those in the IVUS-preferred group (IVUS: 36.3±5.0 mm, CT: 32.9±5.1 mm; p = 0.030). All stent grafts were successfully implanted (100%). IVUS did influence the stent graft choice in 44% (n = 15) of the implanted Valiant/Relay stent grafts; in the GORE group, IVUS leaded to different stent graft choice in 5 times (45%, Fig 3).

**Table 2:** Stent grafts used for thoracic endovascular aortic repair.

	IVUS *n* = 20	CT *n* = 25	Total
Valiant (Medtronic), *n (%)*	9 (45)	8 (32)	17
Relay (Bolton), *n (%)*	6 (30)	11 (44)	17
GORE (Gore), *n (%)*	5 (25)	6 (24)	11

IVUS, intravascular ultrasound; CT, computed tomography.

### Aortic remodeling in both groups

**Fig 4:**
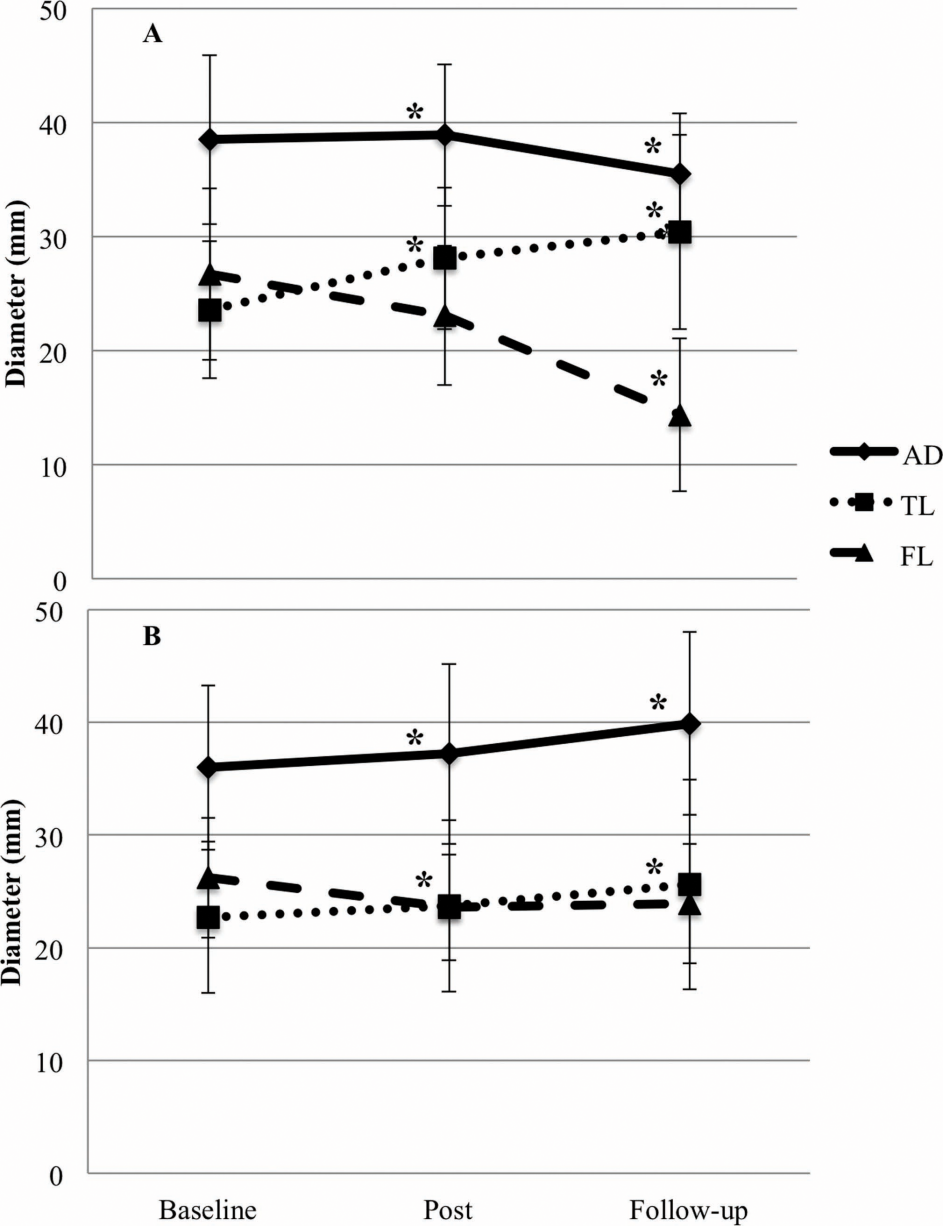
Aortic remodeling depending on sizing strategy. Mean diameters and standard deviation for true lumen (TL), false lumen (FL) and total aorta (AD) are shown separately for the IVUS-guided (A) and CT-guided (B) group at baseline, one day after implantation (post) and follow-up. * differs significantly from baseline.

The mean follow-up time was 24.4±22.8 months (median 16.8 months) in the collective group, with a minimum of 1.5 months and a maximum of 79.9 months. The follow-up times between the IVUS-preferred and the CT-preferred groups did also not differ significantly (IVUS 22.9±23.1 months, CT 25.6±23.0 months; *p* = 0.700). The assessed diameters of baseline measurements in both groups were comparable for TL, FL, and AD. With regard to post-treatment control, the TL exhibited significant expansion in the IVUS group, whereas diameters for FL and AD did not differ significantly between the two groups. This trend was still evident at follow-up, while FL and AD were significantly reduced in the IVUS group (Table 3, Fig 4).

**Table 3:** Measurements of true lumen (TL), false lumen (FL) and aortic diameter (AD) in both groups at the distal stent end (DSE), pulmonary artery (PA) and diaphragm (DP).

	IVUS *n* = 20	CT *n* = 25	*p* value
TL pre-treatment, DSE	24.3±5.1	22.4±4.4	0.180
TL post-treatment, DSE	28.9±5.8	24.2±4.2	0.002*
TL follow-up, DSE	31.4±5.8	26.2±4.8	0.002*
TL follow-up, PA	31.5±4.2	27.5±4.4	0.004*
TL follow-up, DP	30.9±7.2	25.5±5.7	0.008*
FL pre-treatment, DSE	27.2±5.0	26.2±6.3	0.564
FL post-treatment, DSE	25.6±5.9	23.3±7.2	0.259
FL follow-up, DSE	16.3±11.6	23.9±8.3	0.014*
FL follow-up, PA	16.2±8.1	20.9±10.7	0.045*
FL follow-up, DP	17.6±7.3	25.3±8.7	0.003*
AD pre-treatment, DSE	38.3±7.5	35.9±6.7	0.266
AD post-treatment, DSE	38.6±7.3	37.0±7.3	0.245
AD follow-up, DSE	35.2±6.6	39.4±6.9	0.043*
AD follow-up, PA	35.9±6.3	40.8±6.9	0.018*
AD follow-up, DP	35.1±7.1	39.2±6.0	0.045*

Shown are mean-values ± SD (mm). DSE, distal stent end; PA, pulmonary artery; DP, diaphragm; IVUS, intravascular ultrasound; CT, computed tomography; TL, true lumen; FL, false lumen; AD, aortic diameter.

* means significant

A subanalysis of acute aortic dissections (n = 19 vs. n = 13) showed the same tendency of aortic remodeling: At the follow-up, the size of TL expanded significantly in the IVUS group (CT, n = 13: 25.0 ± 6.1 mm; p = 0.029), whereas FL and AD were significantly reduced in the IVUS group (CT, n = 13: 24.2 ± 9.6 mm; p = 0.007 and 41.0 ± 8.5 mm; p = 0.049, respectively). Several outcome data is presented in Table 4. Notably, IVUS did not change the re-intervention rate significantly, but we found a longer time frame free from re-intervention compared to the CT group.

**Table 4:** Related outcome characteristics after TEVAR at follow-up.

	IVUS *n* = 20	CT *n* = 25	*p* value
Type I endoleak	1 (5)	1 (4)	0.872
Totally remodeling	2 (10)	0 (0)	0.106
Dead	2 (10)	2 (8)	0.815
Re-intervention	5 (25)	11 (44)	0.186
Time to re-intervention	25.5±16.7	11.3±7.3	0.029*

Shown are mean-values ± SD (months) or n (%). TEVAR, thoracic endovascular aortic repair.

* means significant

## Discussion

While dilatation of the aortic diameter increases the risk of rupture in Type B aortic dissection and is associated with impaired outcome, the optimal sealing of the proximal entry tear is thought to induce better aortic remodeling by diverting the blood flow through the true lumen and leading to shrinkage of the false lumen and total aortic diameter. The accompanied thrombosis of the false lumen has been demonstrated to be a good prognostic indicator [9]. The present study provides insights into the aortic remodeling process after TEVAR in patients with Type B aortic dissection and illustrates the benefit of IVUS-assisted stent graft sizing in TEVAR over time. Due to the tortuosity of the aortic arch and the drift out of the coaxial axis, IVUS tends to overestimate luminal diameter [8,10]. As a high divergence of measurements is particular seen at level of the LSA, divergent results can be challenging when choosing an appropriate stent graft. The current study suggests that if the pitfalls of IVUS are understood and the measurements the bias of IVUS are taken into account, IVUS-assisted sizing in TEVAR is valid, even with results that vary from diameters measured via CT. Notably, the differences between IVUS and CT measurements at the level of the LSA were still significant, even after sub-dividing the subjects into the IVUS-assisted and CT-assisted groups. Acute aortic dissection had been detected in the majority of the IVUS group, and in that group significantly more patients underwent emergency TEVAR. This condition in particular may be associated with a specific strength of IVUS-pitfalls in CT angiography may result in difficulties in distinguishing aortic borders, TL, and FL [11]. IVUS offers valuable information regarding the underlying aortic disease with precise localization of significant pathologies in real-time, and therefore may have an important impact on treatment in these specific circumstances [12,13]. Moreover, IVUS assistance is of an additional value in patients with impending or already existing aortic rupture, especially after blunt trauma where the accurate estimation of the vessel diameter at the time of CT is difficult due to poor volume filling. IVUS was already shown to be used in patients with such impaired hemodynamic conditions [14]. IVUS enables the measurement of the exact diameter quite before TEVAR after stabilization of the patient with volume filling and reduces the incidence of oversizing of the stent graft. There is no firm consensus about the correct sizing of stent grafts in these patients [15] and remaining differences in luminal diameter at the time of TEVAR compared with initial CT are still challenging and affect the majority of intended graft sizes [16]. IVUS provides real-time assessment of the aortic diameters directly before TEVAR with exact identification of proximal and distal landing zone. If the CT is of poor imaging quality, IVUS may deliver exact measurement in these regions of interest and help guiding the intervention in real-time. However, the tendency of overestimating luminal diameters in IVUS, especially in the aortic arch, has to be taken into account. The potential mismatch between a true lumen and a stent graft might continue down the descending aorta, resulting in distal oversizing with a risk of distal stent graft-induced new entry, a rare but potentially fatal complication [17,18]. For the assessment of potential long-term benefits of IVUS-assisted stent graft sizing, we evaluated the aortic remodeling over time. Sealing of the proximal entry tear using TEVAR is thought to redirect the blood flow to the TL immediately. In the current study, we observed the expansion of TL after TEVAR in both groups, which is concordant with previous studies [19]. With regard to follow-up results, IVUS-assisted TEVAR yielded a greater gain in luminal diameter over time than CT, which ultimately is associated with more optimal sealing. The superiority of IVUS assistance for early expansion of the TL after TEVAR was detected. In contrast, the reduction in FL and AD was similar in both groups, and was detected later. In this respect, our analysis may be the first to provide detailed insights into the beneficial effects of IVUS-assisted stent graft sizing in TEVAR on aortic morphology during long-term follow-up.

## Conclusion

IVUS-assisted stent graft sizing in Type B aortic dissection shows beneficial effects on aortic remodeling, and emphasizes the additional advantage of IVUS in aortic diseases. While IVUS will not replace CT as a state-of-the-art technique for stent graft sizing in TEVAR, it may be a valid additional approach, especially when assessment via CT is compromised by poor image quality or is otherwise inconclusive. It may reduce the need for repetition of previous external CT scans of low quality for accurate stent-graft sizing and after TEVAR it can further ensure the apposition of the stent-graft to the aortic wall both to improve aortic remodeling.
